# Sex differences in the experience of COVID-19 post-traumatic stress symptoms by adults in South Africa

**DOI:** 10.1186/s12888-022-03883-6

**Published:** 2022-04-04

**Authors:** Ntombifuthi P. Nzimande, Maha El Tantawi, Roberto Ariel Abeldaño Zuñiga, Richmond Opoku-Sarkodie, Brandon Brown, Oliver C. Ezechi, Benjamin S. C. Uzochukwu, Passent Ellakany, Nourhan M. Aly, Annie Lu Nguyen, Morenike Oluwatoyin Folayan

**Affiliations:** 1 Mental Health and Wellness Study Group, Ile-Ife, Nigeria; 2 Department of Economic and Social Geography, University of Szeged, Szeged, Hungary; 3 Faculty of Dentistry, Alexandria University, Alexandria, Egypt; 4 Postgraduate Department, University of Sierra Sur, Oaxaca, Mexico; 5 Doctoral School of Mathematics, University of Szeged, Szeged, Hungary; 6 Center for Healthy Communities, Department of Social Medicine, Population and Public Health, UCR School of Medicine, Riverside, California USA; 7 Department of Clinical Sciences, Nigerian Institute of Medical Research, Lagos, Nigeria; 8 Department of Community Medicine, College of Medicine, University of Nigeria Nsukka (Enugu campus), Nsukka, Nigeria; 9 Department of Substitutive Dental Sciences, College of Dentistry, Imam Abdulrahman Bin Faisal University, Dammam, Saudi Arabia; 10 Department of Family Medicine, Keck School of Medicine, University of Southern California, Los Angeles, California USA; 11 Department of Child Dental Health, Obafemi Awolowo University, Ile-Ife, Nigeria

**Keywords:** COVID-19, South Africa, Sex, Post-traumatic stress symptoms

## Abstract

**Background:**

The COVID-19 pandemic has created multiple mental health challenges. Many residents in South Africa face pre-existing elevated levels of stress and the pandemic may have had varying impacts on sub-populations. The aims of this study were to determine: 1) the factors associated with post-traumatic stress symptoms (PTSS) and 2) sex differences in the factors associated with PTSS in adults residing in South Africa during the COVID-19 pandemic.

**Methods:**

Study participants aged 18 years and above, were recruited for this cross-sectional study through an online survey implemented from June 29, 2020 to December 31, 2020. The outcome variable was PTSS; explanatory variables were sex at birth, COVID-19 status, social isolation and access to emotional support. Confounders considered were age, education level completed and current work status. Logistic regressions were used to determine the association between the outcome and explanatory variables after adjusting for confounders.

**Outcomes:**

There were 489 respondents. Among all respondents, those who were older (AOR: 0.97; 95% CI: 0.95 – 0.99) and had access to emotional support from family and relatives (AOR: 0.27; 95% CI: 0.14 – 0.53) had significantly lower odds of PTSS. Respondents who felt socially isolated had higher odds of PTSS (AOR: 1.17; 95% CI: 1.08 – 1.27). Females had higher PTSS scores and higher odds of PTSS compared to males (AOR: 2.18; 95% CI: 1.41-3.39). Females (AOR: 0.27; 95% CI: 0.08 – 0.95) and males (AOR: 0.26; 95% CI: 0.11, 0.59) who had access to emotional support had significantly lower odds of PTSS than those who had no support. Females (AOR: 1.15; 95% CI: 1.04 -1.27) and males (AOR: 1.19; 95% CI: 0.11, 0.59) who felt socially isolated had higher odds of PTSS compared to those who did not feel socially isolated.

**Interpretation:**

Compared to males, females had higher scores and higher odds of reporting PTSS during the COVID-19 pandemic. Access to emotional support ameliorated the odds of having PTSS for both sexes, while feeling socially isolated worsened the odds for both sexes.

## Introduction

The coronavirus infectious disease 2019 (COVID-19) had varied effects on different populations. Environmental factors that existed before COVID-19 can contribute to population disparities in terms of the impact of the pandemic. In South Africa, institutionalised racial segregation, known as apartheid, and the violent struggle to dismantle this system in an effort to ensure South Africa is a liberated, democratic society contributes to stressful living. The high prevalence of violent crime resulting from widening socio-economic gaps also contributes to daily stress that may result in complex post-traumatic stress disorders (PTSD) [1]. Among the general population, 73.8% have lifetime exposure to at least one potentially traumatic event [1]. South Africa also has the highest global prevalence of HIV and tuberculosis – infectious diseases that are associated with increased risk for mental health disorders [2].

Stress and trauma resulting from the COVID-19 pandemic will likely exacerbate pre-existing mental health challenges among South Africans or contribute to the development of mental health disorders among healthy citizens [3]. Infectious disease outbreaks, such as Hemagglutinin Type 1 and Neuraminidase Type 1 (H1NI) influenza and Ebola were associated with significant detrimental, long-term psychosocial complications [4]. The stigmatisation, social distancing or isolation restrictions, effect of quarantine, spread of fake news and general frustration related to the pandemics were associated with a range of psychiatric comorbidities such as anxiety and depression [5, 6].

As detailed elsewhere [7], COVID-19, caused by the severe acute respiratory syndrome coronavirus 2 (SARS-CoV-2), is a highly transmissible disease that emerged in late 2019 in Wuhan, China. South Africa detected its first positive COVID-19 case on the 5th of March in 2020 and a national directive was issued for a 21-day national lockdown starting on the 27th of March which gradually eased from 30 April to 18 September in five alert levels. The lockdown in South Africa was among the world’s strictest and earliest lockdowns. Citizens were allowed to leave their homes only for essential services, to seek emergency or chronic medical services, and to collect social grants [8]. While the stringent restriction measures may have enabled South Africa to keep its COVID-19 infection rate lower than anticipated and help it fast-track its return to routine life, the pandemic and the response may have taken a toll on the mental health of the citizens [9].

Illness, self-isolation, quarantine and lack of support structures are just a few pandemic-driven stressors that may have been caused by the COVID-19 pandemic and these stressors may contribute to post-traumatic stress symptoms (PTSS). Unlike post-traumatic stress disorder (PTSD), PTSS is not clinically diagnosed, and is understood as a normal and adaptive response to experiencing of a traumatic or stressful event [10]. Like PTSD, PTSS is associated with feelings of fear, nervousness, avoiding activities or places associated with the traumatic event, and nightmares. The intensity and duration of the symptoms are, however, less severe with PTSS when compared with PTSD [10]. The outbreak of an infectious disease such as COVID-19, is a traumatic experience with direct and indirect impacts on the individual.

COVID-19 had significant impact on the mental health on the population of South Africa [3]. Its impact on the mental health and wellbeing of adults in South Africa might vary between sexes. Several studies on the effect of the pandemic show that females are at higher risk than males for developing mental health conditions [6, 11–14]. A study in China found that female participants had higher prevalence of PTSS compared to their male counterparts [12]. Similarly, a study conducted in Nigeria indicated than non-male gender was associated with higher symptoms of PTSS during the COVID-19 pandemic [15]. However, a different study conducted in Nigeria reported a non-significant impact of gender on PTSS even though the overall prevalence of psychological distress increased during the pandemic [13].

In South Africa, the unexpected death of a loved one and witnessing trauma on others were the most common traumatic events associated with PTSD [16]. Experts in South Africa have predicted that the number of PTSD cases are likely to increase during the pandemic due to numerous socio-economic and psychological factors such as social distancing, isolation, and anxiety [6, 8]. A recent report by the South African Depression and Anxiety Group and Childline confirmed this prediction [17]. There also may be sex disparities in the factors associated with mental health disorders. These socio-economic and psychological factors are associated with increased prevalence of depressive symptoms associated with the pandemic, and higher among females compared to males (28.5% vs. 24.4%) [14].

The aims of this study were to determine: 1) the factors associated with post-traumatic stress symptoms (PTSS) and 2) sex differences in the factors associated with PTSS in adults residing in South Africa during the COVID-19 pandemic. We hypothesized the proportion of respondents with PTSS will differ by sex.

## Methods

### Study design and study population

This was a cross-sectional, multi-country study that recruited study participants globally using an online electronic survey (SurveyMonkey) accessible to participants from 29 June to 31 December of 2020. The survey was designed to determine the impact of COVID-19 on the mental health and wellness of adults using a questionnaire that was initially developed for a study that targeted a specific population in the United States and was consequently adapted and validated for use by a global audience [18]. The questionnaire underwent four iterative processes of content validation prior to global implementation. The overall content validity index was 0.83. Responses collected for content validation were excluded from the final analysis.

Study participants were individuals aged 18 years and above who provided consent to participate in the online survey. The questionnaire was preceded by a brief introduction explaining the purpose of the study, and assuring participants of their voluntary participation and confidentiality of their data. The questionnaire took an average of 11 min to complete and was administered in English. Each participant could only complete a single questionnaire though they could edit their answers freely until they choose to submit. The study had no exclusion criteria.

### Recruitment of study participants

Participants were recruited through respondent driven and snowball sampling. Initial participants were asked to share the survey link with contacts within their countries to facilitate recruitment. The survey link was also posted on social media groups (Facebook, Twitter, and Instagram) and network email lists and WhatsApp groups of initial participants. Study participants were asked to complete an anonymous, closed-ended questionnaire.

### Sample size

A total of 17,008 participants participated in the global study. Data from a sample of participants residing in South Africa (*N* = 498) during the COVID-19 pandemic were extracted for the current analyses.

### Sociodemographic variables

Data included age, education completed (no formal education, primary, secondary, university), current work status (employed, unemployed student), and sex assigned at birth (male, female, intersex, decline to answer).

### The post-traumatic stress symptoms

The Post Traumatic Stress Disorder Checklist for civilians (PCL-C), a 17-item, self-report questionnaire, was used to measure symptoms of PTSD which is based on the Diagnostic and Statistical Manual of Mental Disorders, Fourth Edition (DSM-IV) criteria [19]. Respondents indicated the level of agreement with a litany of problems or complaints in response to a stressful life experience over the past month because of the COVID-19 pandemic on a 5-point scale (1- not at all to 5- extremely). The possible scores ranged from 17 to 85. Higher total scores indicate greater risk for PTSD. The cut-off point was established at 44 to differentiate between no indication and some indication of PTSS [20]. The PCL-C consists of three subscales that correspond to the DSM-IV symptom clusters: re-experiencing, avoidance/numbing, and arousal. The Cronbach alpha for respondents from South Africa based on data for the current study was 0.939.

### Social isolation

Respondents were asked to respond to a question inquiring about their feeling of social isolation on a scale of 1 - not at all - to 10 - extremely. The question was adopted from the study tool utilised by Nguyen et al. [18].

### Emotional support

The Pandemic Stress Index is a 3-item measure. One of the measures assesses access to emotional support from friends and family. A check on this item indicated access to support [20].

### COVID-19 status

Respondents were asked if they knew someone who died from COVID-19 (yes/no). Respondents were also asked how much is/did COVID-19 negatively impact your day-to-day life? The response options were ‘extremely’, ‘very much’, ‘much’, ‘a little’ and ‘not at all’. The responses were dichotomised to ‘yes, had negative impact’ (extremely, very much, much, a little) and ‘no negative impact’ (not at all) [18].

### Statistical analysis

Descriptive analyses of all variables was conducted using SPSS version 23. PTSS (yes versus no) was the outcome variable. The explanatory variable was sex at birth. T-test was used to compare quantitative variables while the chi-square was used for categorical variables between the groups PTSS (yes/ no). The gender differences in the mean scores of the three subscales of the PCL-C (re-experiencing, avoidance/numbing, and arousal) were also determined. Multivariable logistic regression was used to assess the relationship between the outcome and explanatory variables. Confounding variables were age, employment status, and education level of the participants. Covariates were COVID-19 impact on life, feeling of social isolation, access to emotional support from family and knowing someone who died from COVID-19. Stratified analyses by sex were performed.

#### Ethical considerations

Ethical approval of the current study was obtained from the Human Research Ethics Committee at Institute of Public Health of the Obafemi Awolowo University Ile-Ife, Nigeria (HREC No: IPHOAU/12/1557) as the lead partner for this study. All methods were carried out in accordance with international research guidelines.

## Results

There were 489 respondents from South Africa with a mean age of 30.8 years (SD = 9.5). Of the 489 respondents, 173 (35.4%) reported the presence of PTSS. Respondents with PTSS were significantly younger than those without symptoms (29.3 years (SD = 7.7) vs 32.1 years (SD = 10.7); *p* = 0·001), Table 1 displays the factors associated with PTSS in bivariate analyses. Significantly more respondents who were unemployed (*p* = 0.048), female (*p* <  0.001), who felt socially isolated (*p* <  0.001) and who reported that COVID-19 has a negative impact on their daily life (*p* = 0.005) had PTSS. PTSS was more frequent among participants reporting less emotional support from family and friends (*p* < 0.001).

**Table 1:** Factors associated with post-traumatic stress symptoms in South Africa residents during the COVID-19 pandemic (*N* = 489)

Variables	Post-traumatic stress symptoms		***p*** value	Total *N* = 489
	Absent *N* = 316	Present *N* = 173		
	*n* (%)	*n* (%)		*n* (%)
**Sex at birth**				
Male	137 (43·0)	43 (24·9)	< 0·001	179 (36·6)
Female	179 (56·6)	130 (75·1)		309 (63·2)
Decline to answer	1 (0·3)	0 (0)		1 (0·2)
**Age**				
Mean (SD)	32.1 (10.7)	29.3 (7.7)	0.001	30.8 (9.5)
**Educational level**				
Less than university education	36 (11·4)	24 (13·9)	0·42	60 (12·3)
University education	280 (88·6)	149 (86·1)		429 (87·7)
**Work Status**				
Employed	181 (57·3)	83 (48·0)	0·048	264 (54·0)
Unemployed	135 (42·7)	90 (52·0)		225 (46·0)
**Social isolation**				
Mean (SD)	5.18 (2·61)	6.43 (2·74)	< 0·001	5.62 (2·72)
**Get enough emotional support from family and relatives**				
Yes	301 (95·3)	140 (80·9)	< 0·001	441 (90·2)
No	15 (4·7)	33 (19·1)		48 (9·8)
**Know someone who died from COVID-19**				
Yes	142 (44·9)	86 (49·7)	0·31	228 (46·6)
No	174 (55·1)	87 (50·3)		261 (53·4)
**COVID-19 has negative impact on daily life**				
Yes	261 (82·6)	159 (91·9)	0·005	420 (85·9)
No	55 (17·4)	14 (8·1)		69 (14·1)

Table 2 shows gender differences on the three subscales of the PCL-C. Females had significantly higher scores than males on all three subscales (re-experiencing, avoidance and hyperarousal) as well as the total PCL-C score.

**Table 2:** Gender differences in the PCL-C sub-scales re-experiencing, avoidance, and hyperarousal (*N* = 489)

PTSD criteria (DSM-IV)	Gender	N	Mean	Standard deviation	*p*-value
Re-experiencing	Females	309	11.35	5.46	< 0.001
	Males	180	9.16	4.94	
Avoidance	Females	309	16.66	6.82	< 0.001
	Males	180	14.11	6.69	
Hyperarousal	Females	309	13.13	5.32	< 0.001
	Males	180	10.57	5.11	
PCL-C total score	Females	309	41.15	15.72	< 0.001
	Males	180	33.84	15.56	

Table 3 shows that sex, age, social isolation and emotional support were significantly associated with PTSS. Females (AOR: 2.19; 95% CI: 1.41-3.39; *p* < 0.001) had higher odds of PTSS than males. Also, respondents who felt more socially isolated had higher odds of PTSS (AOR: 1.17; 95% CI: 1.08 – 1.27; *p* < 0·001). Respondents who were older (AOR: 0.97; 95% CI: 0.95 – 0.99; *p* < 0.039) and who felt they had enough emotional support from family and relatives (AOR: 0.27; 95% CI: 0.14 – 0.53; *p* < 0.001) had lower odds of PTSS.

**Table 3:** Multivariate logistic regression analysis for factors associated with PTSS among residents of South Africa during the COVID-19 pandemic (*N* = 489)

Variables	AOR (95% CI)	***p***-value
**Sex**		
Male	1.00 (–, –)	< 0·001
Female	2·19 (1·41, 3·39)	
**Age**	0·97 (0·95, 0·99)	0·04
**Education level**		
University education	1.00 (–, –)	0·88
Less than university education	1·05 (0·56, 1·97)	
**Employment status**		
Employed	1.00 (–, –)	0·41
Unemployed	0·83 (0·54, 1·29)	
**Social isolation (0-10-point scale)**	1·17 (1·08, 1·27)	< 0·001
**Get enough emotional support from family and relatives**		
No	1.00 (–, –)	< 0·001
Yes	0·27(0·14, 0·53)	
**Know someone who died from COVID-19**		
No	1.00 (–, –)	–
Yes	0·89 (0·59, 1·34)	0·058
**COVID-19 impact on day-to-day life**		
Yes	1.00 (–, –)	0·08
No	0·55 (0·28, 1·08)	

*AOR* Adjusted Odds Ratio

*p*-value< 0·05

Table 4 shows that there are only two factors associated with sex differences in PTSS: social isolation and access to emotional support. The greater the perception of social isolation, the higher the odds of PTSS in both female (AOR: 1.15; 95% CI: 1.04 -1.27) and male (AOR: 1.19; 95% CI: 1.03-1.38) respondents. Also, female (AOR: 0.27; 95% CI:0.08 – 0.95) and male (AOR: 0.26; 95% CI: 0.11- 0.59) respondents who received emotional support from family and relatives had lower odds of PTSS.

**Table 4:** Multivariate logistic regression analysis for factors associated with sex differences in PTSS among residents of South Africa during the COVID-19 pandemic (*N* = 489)

Variables	Female		Male	
	AOR (95% CI)	***p***-value	AOR (95% CI)	***p***-value
**Age**	0·98 (0·95, 1·01)	0·190	0·96 (0·91, 1·01)	0·088
**Educational level**				
University	1.00	–	1.00	–
Less than university	0·96 (0·48, 1·95)	0·918	1·27 (0·32, 5·10)	0·738
**Employment status**				
Employed	1.00	–	1.00	–
Unemployed	0·75 (0·44, 1·26)	0·276	1·04 (0·46, 2·39)	0·919
**Social isolation**				
(0-10 point scale)	1·15 (1·04, 1·27)	0·004*	1·19 (1·03, 1·38)	0·018
**Get enough emotional support from family and relatives**				
No	1.00	–	1.00	0·041*
Yes	0·27 (0·08, 0·95)	0·001*	0·26 (0·11, 0·59)	
**Know someone who died from COVID-19**				
No	1.00	–	1.00	0·611
Yes	0·92 (0·57, 1·50)	0·751	0·82 (0·39, 1·74)	
**COVID-19 impact your day-to-day life**				
Yes	1.00	–	1.00	0·161
No	0·62 (0·29, 1·33)	0·221	0·33 (0·07, 1·55)	

Note: *AOR* Adjusted Odds Ratio, *CI* Confidence Interval

^*^statistically significant at *p* value < 0·05

## Discussion

Findings indicated sex differences for PTSS during the COVID-19 pandemic. Compared to males, females had significantly higher odds of PTSS as well as significantly PTSS scores and higher re-experiencing, avoidance/numbing, and arousal scores during the pandemic. The findings supported the study hypothesis that PTSS would manifest at different rates by sex. Findings also show that for both females and males, feeling socially isolated was associated with higher odds for PTSS while older age and access to emotional support from friends and families were associated with lower odds of PTSS.

One of the strengths of the study is its contribution to the growing knowledge of sex disparities in mental health during the COVID-19 pandemic. The study however, has a few limitations. First, this is cross-sectional study and causal relationships cannot be drawn. Second, although results show sex disparities in PTSS, there were more females than males in the study sample thereby introducing sampling imbalance which may affect the conclusions. Also, the sample size is small and limit the generalizability to all adults in South Africa. Finally, the study used a convenience sample where respondents were those who had access to internet. One can also note that respondents were highly educated (88% had university level) thus the identified factors associated with PTSS among this group may differ from that of other groups. This may have introduced some selection bias. Despite these limitations, the study provides evidence on the effect of the pandemic on the mental health of communities in Africa.

The observed increased odds of PTSS among females compared to males is consistent with results of earlier studies that indicated that marginalised groups (including females) showed extreme psychological distress during the early stages of the pandemic [7, 21] Similar to current findings, being female was found to be associated with a higher level of PTSS in Italy [22], Lebanon [23] and other populations [24, 25]. However, it is essential to note that PTSS is likely not related solely to COVID-19 as it is possible that females were exposed to other traumatic events. This has been supported by Breslau [26] who found that after experiencing a traumatic event, women had a twofold increase in risk for PTSD compared to men. Prior studies have posited that sex differences in PTSS may be due to factors such as females facing higher psychological stress due to increased care burden at home [27]. There are also possible sex differences in behavioural responses to distress and the experience/expression of distress [28]. Ovarian hormone fluctuations and endogenous estradiol changes across the menstrual cycle may also increase susceptibility to stress [29]. On the other hand, males tend to have more pronounced emotion and stress regulation, self-referential processing, and cognitive control thereby lowering their perceived need for psychological support [30]. Sex differences in coping styles in response to stress have also been reported with females being more likely than males to report a lack of alternative coping strategies [31]. Sex differences in PTSS has also been explained by genetic predisposition with females being more likely to inherit anxiety-related risk factors [32]. These explanations may also be associated with females reporting higher levels of loneliness during the pandemic when compared to their male counterparts [33].

The proportion of residents in South Africa who reported PTSS in this study was higher than the 7% reported in China [12], and the pre-COVID-19 reports of 8 and 14% PTSS in populations in South Africa [34, 35]. The prevalence is however, lower than the 47.5% reported in Nigeria in a study analysed from the same primary dataset used for this study [15]. The high prevalence of PTSS is a concern as this may persist over a relatively long period of time. Risk factors are not only gender related but also include individual, family, societal and pandemic-related factors [36]. Early screening for PTSS and timely evidence-based interventions and social support can mitigate COVID-19 pandemic PTSS [37].

The finding that younger participants had higher odds of exhibiting PTSS during the COVID-19 pandemic is consistent with prior studies that indicated similar relationships before the pandemic [36] and contrary to others that had reported a direct relationship between age and PTSD before the pandemic [38]. Older age is a stronger risk factor for PTSD for males than it is for females [5, 39], but PTSD was reported in younger men during the COVID-19 pandemic [7]. The association between ag and PTSS in our study may be a reflection of younger respondents having less access to social support, feelings more socially isolated and the impact of media exposure [40]. The restrictions imposed by the pandemic made it harder for young people to capitalize on social connections, explore social opportunities and networking events to advance careers and job opportunities; these all cause stress for young people [41].

We found that access to emotional support was protective of PTSS while social isolation was a risk indicator. Access to social support influences the mental health consequences of stressful life events by buffering against psychological distress [42]. Social isolation has been associated with increased heart rate and blood pressure and hypercortisolemia [43] while access to social support foster resilience and reduce the risk for developing mental illness [44]. The long-term detrimental effect of feeling socially isolated during the pandemic necessitates the need for screening, identifying and providing support for persons who feel isolated. The South African government together with key stakeholders instituted initiatives to mitigate harm to the mental health of the general population by setting up several free hotlines through which its citizens can contact a professional staff to assist with mental health issues. These avenues include the South African Depression and Anxiety Group Helpline and the Cipla Foundation WhatsApp helpline [45], amongst others. However, it is important to monitor and evaluate how the helplines have helped to ameliorate the impact of the pandemic on the risk for PTSS by the population.

The study findings have policy implications. First, priority attention need to be given to females and those who feel socially isolated during the pandemic because they are more likely to experience PTSS, which has possible cascades like neurological conditions, substance use, mood disorders and other anxiety disorders [46]. Screening for PTSS as part of routine health care services may enable the government to promptly identify persons who may need support, and facilitate their access to emotional support from friends and families where feasible. Further studies are needed to explore the findings of this study, including identifying effective behavioural and coping strategies for the people living in South Africa.

## Concluding remarks

Though we observed that females had higher PTSS scores and higher odds of PTSS than men, factors that modify the risk for PTSS may be similar for both sexes as indicated by the results of this survey. Screening of females for PSTD and symptoms related to PTSD during the COVID-19 pandemic may facilitate early detection and prompt treatment. Females should also be supported with PTSD prevention through the use of multiple health service outlets that provide accessible services for females.

## Data Availability

The datasets used and/or analysed during the current study are available from the corresponding author on reasonable request.

## Abbreviations

AOR: Adjusted Odds Ratio
CI: Confidence Interval
COVID-19: Coronavirus Infectious Disease
PCL-C: Post Traumatic Stress Disorder Checklist for civilians
PSTD: Post Traumatic Stress Disorder
PTSS: Post Traumatic Stress Symptoms
SD: Standard Deviation
